# Repeated Administration of Baclofen Modulates TRPV-1 Channel Expression by PKC Pathway in Dorsal Root Ganglia of Spinal Cord in a Morphine Tolerance Model of Rats

**DOI:** 10.29252/ibj.24.6.374

**Published:** 2020-06-21

**Authors:** Shima Mehrabadi, Seyed Morteza Karimiyan, Ghorbangol Ashabi, Khadijeh Moradbeygi, Marjan Hoseini

**Affiliations:** 1 Department of Physiology, School of Medicine, Tehran University of Medical Sciences, Tehran, Iran;; 2 Department of Nursing, Abadan Faculty of Medical Sciences, Abadan, Iran

**Keywords:** Baclofen, Morphine, Protein kinase C, Spinal cord

## Abstract

**Background:**

Tolerance and dependence to anti-nociceptive effect of morphine restricted its use. Nowadays co-administration of morphine and other drugs suggests diminishing this tolerance. Baclofen is one of the drugs that may be beneficial in the attenuation of tolerance to morphine. Studies have shown that changes in TRPV-1 expression during administration of morphine have a pivotal role in developing morphine tolerance. Therefore, the effect of baclofen on TRPV-1 expression during chronic administration of morphine was investigated in this study.

**Methods:**

A total of 48 rats were divided into four groups of control, morphine single injection, morphine tolerance, and morphine tolerance + baclofen. To induce morphine tolerance in rats, animals received 10 mg/kg of i.p. morphine sulfate once a day for 10 days. In the treatment group, baclofen (0.5 mg/kg) was injected for 10 days, before morphine injection. Finally, to evaluate baclofen treatment on morphine analgesia and hyperalgesia, thermal hyperalgesia and formalin test were used. TRPV-1 and PKC expression and protein production in DRG of spinal cord were then evaluated by real-time PCR and Western blot.

**Results:**

In baclofen treatment group, thermal hyperalgesia and formalin test improved in comparison with morphine tolerance group. In morphine tolerance group, both TRPV-1/PKC gene expression and protein levels increased in comparison with the control group. However, following the baclofen treatment, the TRPV-1 and PKC levels decreased.

**Conclusion:**

Baclofen can enhance anti-nociceptive effect of morphine by modulating TRPV-1 channel and PKC activity.

## INTRODUCTION

Opioids are the most common analgesic drugs applied in clinical practice to treat chronic pain^[^^1^^]^. As a MOR agonist, morphine is used extensively to attenuate chronic pain, but morphine tolerance, dependence, and addiction restrict its administration^[^^2^^,^^3^^]^. Nowadays, co-administration of morphine plus other drugs suggests diminishing this tolerance^[^^4^^-^^6^^]^. As a GABA_B_ agonist, baclofen might be beneficial to minimize morphine tolerance^[^^7^^,^^8^^]^. Recently, behavioral and molecular studies have shown that baclofen could be used along with opioid drugs to delay tolerance and dependence to opioids^[^^3^^,^^9^^]^. Evidence has also indicated that baclofen may potentiate morphine analgesia in chronic use of opioids and can reduce many adverse effect of chronic administration of opioids^[^^6^^,^^10^^]^. Earlier investigations have reported that GABA_B_ receptor agonist decreases the cocaine and nicotine self-administration^[^^11^^,^^12^^]^. Opioid tolerance is a brain disorder. Although many researches have been conducted to diminish this tolerance; the majority has been unsuccessful^[^^13^^]^. 

TRPV1 is responsible for pain perception^[^^14^^]^ and is known as a sensory ion channel that transduces a harmful environment stimulant into electrical depolarization^[^^15^^]^. TRPV1 is expressed in the central nervous system, especially in the dorsal horn of spinal cord and plays an important role in pain transmission^[^^16^^]^; however, its function in opioid dependence has not been studied well. There are many signaling pathways activated or inhibited by TRPV1. Many studies have demonstrated that the modulation of TRPV1 involves triggering many cascades of signal transduction^[^^17^^-^^19^^]^. TRPV1 activation in spinal cord can result in the stimulation of N-type and T-type voltage-gated Ca^2+^ channels, which in turn activate many isoforms of PKC^[^^1^^,^^20^^]^. 

PKC activation can also trigger many molecules in signal transduction pathways and alters the function of sensory neurons in DRG^[^^21^^,^^22^^]^. Surveys have also indicated that PKC activation can activate more TRPV1 channels^[^^23^^,^^24^^]^. In a study, it was confirmed that PKC can stimulate TRPV-1 expression and protein production in primary afferent neurons and sensitize them as hyper-sensitization, which takes place in the chronic use of morphine^[^^24^^-^^27^^]^. 

The present study investigates the effect of baclofen on chronic administration of morphine and TRPV-1 and PKC gene expression and protein production in DRG to analyze the possible role of TRPV-1 channel in initiating tolerance to morphine analgesia in chronic use.

## MATERIALS AND METHODS

**Animals**

Forty-eight male Wistar rats (weight: 200-250 g) were included in this study and were housed as four animals per cage under a 12-h light/12-h dark illumination cycle in a temperature-controlled room. All the rats had access to food and water *ad libitum*. Rats were randomly divided into four groups (n = 12 per group): (a) control group that recieved i.p. infusion of normal saline as vehicle once a day for 10 days; (b) morphine single injection group with a dose of 10 mg/kg 30 minutes before behavioral studies to approve that this dose can induce analgesia in single dose; (c) morphine tolerance group that received 10 mg/kg i.p. infusion of morphine once a day for 10 days^[^^9^^]^; (d) baclofen treatment group that received baclofen (0.5 mg/kg) infusion for 10 days before morphine infusion^[^^9^^]^. Ten days following the final injection, behavioral studies were conducted. The rats were then anesthetized with ketamine (90 mg/kg) and xylazine (5 mg/kg) to extract their DRG of spinal cord, which was dissected and frozen in liquid nitrogen and stored at -80 °C for Western blot and real-time PCR studies.

**Morphine tolerance induction**

Morphine hydrochloride (Temad, Iran) was administered at a single 10 mg/kg i.p. dose for 10 days. In treatment group, baclofen (Zahravi Pharmaceutical Co., Iran) was administrated at a 0.5 mg/kg i.p. dose for 10 days. All the drugs were dissolved in physiological saline.

**Formalin test**

Formalin test was used to assess anti-nociceptive effect of morphine on day 10, 30 minutes after the final injection in all groups. The rats were placed in a chamber (40 × 35 cm) with a mirror angled at 45° situated behind the chamber to allow the paws viewed by the observer. First, rats were acclimated in the chamber for 15 min before the experiment began. Following acclimation, formalin (50 μl) was injected subcutaneously into the plantar of left hind paw of rat. After injection, rats were immediately returned to the chamber, and formalin-induced behaviors were recorded by the observer based on pain severity scored from 0 to 3 for 60 min^[^^28^^]^. To analyze the scores, the percentage of area under curve was calculated 0-5 minutes for phase I and 15-60 min for phase II of formalin pain test. 

**Hot plate test**

Hot plate tests were conducted by a hot plate device on day 10, 30 minutes after the final injection in all groups. The rats were placed on a hot plate (50 ± 0.2 °C). Licking, shaking of hind paw, or making an unpleasant sound were considered as the signs of thermal nociception. The interval between being placed on the hotplate and the first signs of thermal nociception was measured. Then the rats were removed immediately when the first sign of thermal nociception was seen by the observer. Delay in showing thermal nociception was recorded as PWT. The baseline latency was 9.7 ± 3.8 s, and cut-off time was 60 s to avoid tissue damage^[^^29^^]^.

**Western blot**

After the extraction of DRG from spinal cord, tissue samples were homogenized in an appropriate buffer and centrifugated at 10000 ×g for 10 minutes. Subsequently, supernatants were collected as lysates. To determine protein concentration in the supernatant, Bradford method was employed. Next, 60 μg protein was loaded on SDS-12.5% polyacrylamide gel electrophoresis as standard lysate and transferred to PVDF membrane (Chemicon Millipore Co. Temecula, USA). Blots were then blocked on an immobilon^TM^-P membrane (Millipore, Bedford, MA, USA). After blocking, the blots were incubated with TRPV1 and PKC primary antibodies (1:1000; Abcam, England) at 4 °C overnight. This process was followed by incubation with rabbit IgG-horseradish peroxidase-conjugated secondary antibody (1:3000; Cell Signaling Technology Co., New York, USA) for 2 h and by detection of reactive bands by chemiluminescence kit reagent (Amersham Bioscience Co. Piscataway, USA). Blots were stripped in a stripping buffer (pH 6.7) and probed with anti β-actin antibody, as an internal control (1:1000; Cell Signaling Technology Co.). The immuno-positive bands were analyzed with ImageJ 1.38 software (National Institutes of Health, Bethesda, MD, USA), normalized to the internal control and expressed as fold changes.

**Real-time PCR**

Real-time PCR was conducted to assess the expression levels of TRPV1 and PKC in brain tissue. Total RNA was extracted by Trizol reagent (DNA biotech Co., Tehran, Iran) and quantified by a nano-drop spectrophotometer (Thermo Fisher, USA). DNase1 (Pishgam, Iran) was used to remove genomic DNA contamination. Afterwards, 1 μg RNA was transcribed reversely by primeScript RT reagent kit (Takara, Japan). Quantitative PCR was performed using SYBER Premix Ex Taq (Takara). A melting curve analysis was conducted following each reaction. The relative mRNA expression was calculated using ΔΔCT method. 

**Data analysis**

GraphPad Prism 7.0 was used to analyze the data, which were represented as mean ± SEM. One-way ANOVA followed by Tukey's post-hoc test was used to compare the results in all studies. Gene expression data were analyzed by Relative Expression Software Tool (REST) Version 2 to determine the difference between the samples and the control group. *p* value <0.05 was considered as statistically significant.

**Ethical statement**

The above-mentioned treatment protocols were approved by the Ethics Committee for the Care and Use of Laboratory Animals at Tehran University of Medical Sciences, Tehran, Iran (ethical code: IR.TUMS.MEDICINE.REC.1397.394). 

## RESULTS

**Effect of co-administration of baclofen plus morphine in formalin test**

**Fig. 1:**
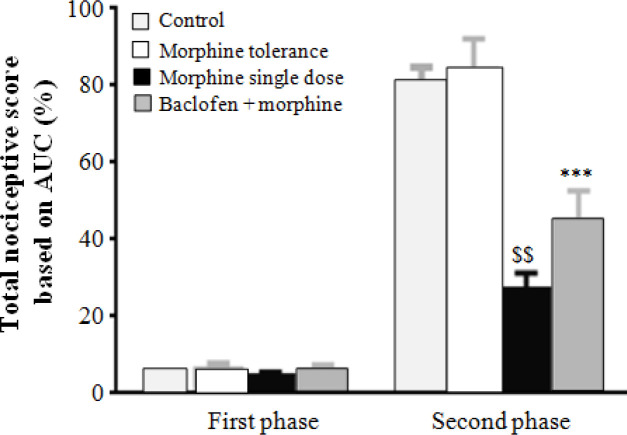
Effect of co-administration of baclofen and morphine in formaline test. Baclofen could decrease the second phase of formalin pain test in morphine tolerance group. Chronic administration of morphine failed to decrease the second phase of formalin pain test in comparison with the control group and induced morphine tolerance in animals. However, a single dose of morphine (10 mg/kg) reduced the second-phase pain of formalin test in comparison with the control and morphine tolerance groups (^$$^*p* < 0.001). Baclofen potentiated morphine analgesic in chronic morphine administration in comparison with morphine tolerance group and decelerated the development of morphine tolerance (^***^p < 0.01). Data were represented as mean ± SEM (n = 10).

Administration of formalin to the left hind paw of rats induced biphasic pain (acute pain: 0-5 min, sever pain: 15-60 min) in the control group. In chronic administration of morphine (10 mg/kg), there was no pain alleviation in formalin pain test in rats. There was also no significant difference between chronic administration in the morphine group and control group. However, administration of a single dose of morphine (10 mg/kg) could provide an analgesic effect on sever pain (second phase) in formalin test in comparison with control and morphine tolerance groups (*p* < 0.0001). A single dose of 10 mg/kg morphine could provide anti-nociception effect on the second phase of formalin test. However, repeated administration of 10 mg/kg morphine developed morphine tolerance in animals. In addition, administration of baclofen (0.5 mg/kg) together with morphine (10 mg/kg) for 10 days could potentiate the analgesic effect of morphine in comparison with the control and morphine tolerance groups (*p* < 0.001) and could prevent morphine tolerance induction in animals (Fig. 1).

**Effect of co-administration of baclofen and morphine in hot plate test**

Results of hot plate test showed that PWT decreased in the morphine tolerance group compared to the control group. Besides, chronic administration of morphine induced hyperalgesia on day 10 in the morphine tolerance group compared to the control group (*p* < 0.05). The single injection of morphine (10 mg/kg) 30 minutes before hot plate test could increase PWT in comparison with the control group (*p* < 0.01).

**Fig. 2:**
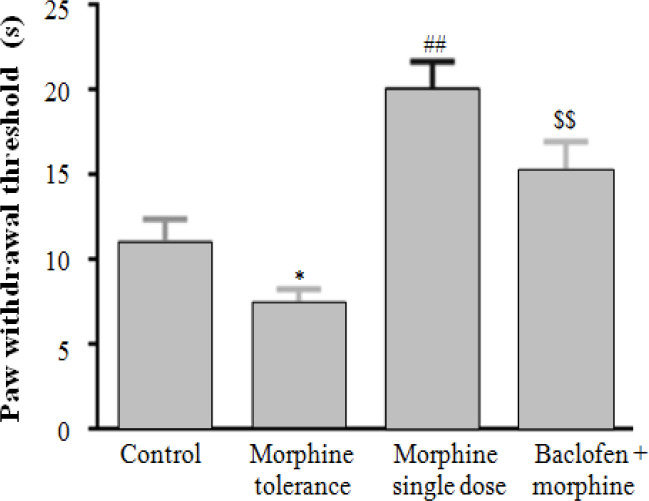
Effect of co-administration of baclofen and morphine in hot plate test. Chronic administration of morphine decreased PWT and induced hyperalgesia on day 10 in the morphine tolerance group compared to the control group (^*^p < 0.05). Single injection of morphine could increase PWT in comparison with the control group (^##^*p* < 0.01). Baclofen plus morphine increased PWT in comparison with the morphine tolerance group (^$$^*p* < 0.01). One-way ANOVA was used for statistical analysis, followed by Tukey's post-hoc test. Data were represented as mean ± SEM (n = 10)

In addition, co-administration of baclofen plus morphine increased morphine analgesic effect; therefore, PWT increased in comparison with morphine tolerance group (*p* < 0.01; Fig. 2).

**Effect of co-administration of baclofen and morphine on TRPV1 channel and PKC protein level**

**Fig. 3:**
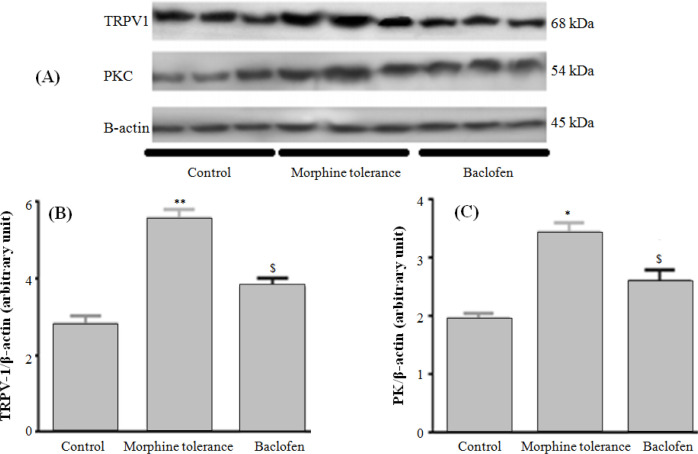
Western blot analysis of TRPV-1 and PKC in DRG in experimental groups. (A) An example of Western blot with PKC and TRPV1 antibody showing bands at 54 kDa and 68 kDa in DRG of morphine-tolerant and control rats. (B and C) Semi-quantitive measurement of Western blot revealed a significant difference in PKC (*p < 0.05) and TRPV1 (**p < 0.01) levels between morphine-tolerant and control rats. Baclofen reduced PKC and TRPV1 levels in morhine tolerant rats (^$^*p* < 0.05). Data were represented as mean ± SEM (n = 6).

To assess the protein level of TRPV1 channel and PKC in DRG, semi-quantitative Western blot analysis was used. Results of analysis (Fig. 3) showed that TRPV1 protein levels increased in DRG of spinal cord in morphine tolerance group in comparison with the control group (*p* < 0.01). Furthermore, PKC production increased in DRG in the morphine tolerance group in comparison with the control group (*p* < 0.05). Co-administration of baclofen and morphine could be effective in reducing TRPV-1 (*p* < 0.05) and PKC (*p* < 0.05) levels compared to the morphine tolerance group (Fig. 3).

**Reduction of TRPV1 channel and mRNA expression of PKC in morphine tolerance group**

Morphine-tolerant rats exhibited a significant elevation in PKC (*p* < 0.05) and TRPV1 (*p* < 0.01) expression levels in DRG compared to the control group. Baclofen administration could significantly reduce PKC (*p* < 0.05) and TRPV1 (*p* < 0.05) levels in comparison with morphine tolerance group. Results of Western blot were consistent with the results of real-time PCR, and the data showed that baclofen plays an important role in the prevention of DRG structure alternation in chronic morphine administration.

**Fig. 4:**
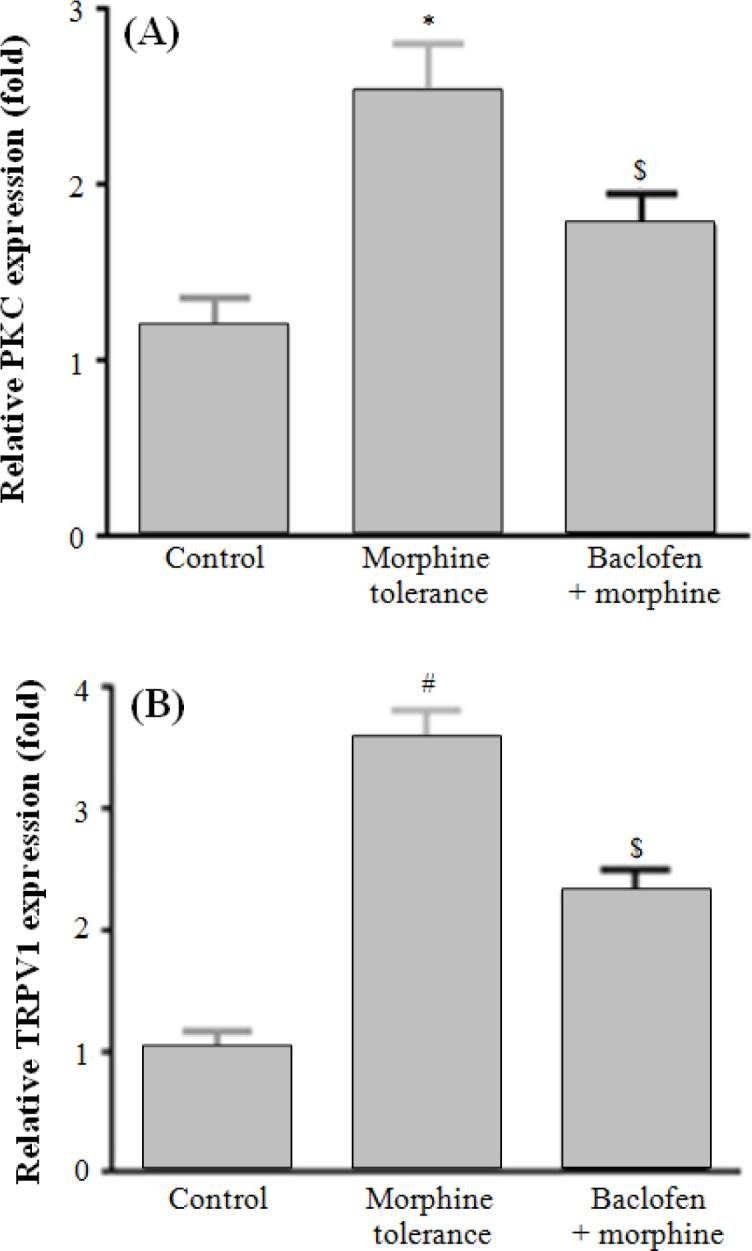
Real-time PCR analysis of TRPV-1 and PKC in DRG in experimental groups. (A) In morphine tolerance group, PKC activity expressed more than the control group (*p < 0.05). Administration of baclofen plus morphine effectively reduced PKC activity in comparison with morphine tolerance group (^$^*p* < 0.05). (B) TRPV1 expression level increased in morphine tolerance group in comparison with the control group (^#^*p* < 0.01). Administration of baclofen plus morphine reduced significantly TRPV1 expression level in comparison to morphine tolerance group (^$^*p* < 0.05). Data were represented as sean ± SEM (n = 4).

## DISCUSSION

This study investigated the effect of baclofen, as a potent GABA_B_ agonist, on the alternation of TRPV1 and PKC expression in DRG of spinal cord in morphine-tolerant rats. Results indicated that the co-administration of baclofen plus morphine could attenuate pain in formalin nociception test as well as in hot plate test, while morphine failed to attenuate pain in both tests. These results show that baclofen can inhibit some alternations and the mechanisms involved in the progression of morphine tolerance. Molecular studies confirmed some changes occurred in morphine tolerance in spinal cord level and how baclofen could prevent these changes. In morphine tolerance development, TRPV1 channel, as an important receptor of pain perception^[^^30^^]^, and PKC, as an important mediator in cells, overexpressed in sensory neurons in the spinal cord^[^^27^^]^. There is also much evidence indicating that TRPV1 activation in the central and peripheral nervous system might be responsible for the development of morphine tolerance and dependence^[^^1^^,^^31^^,^^32^^]^. 

TRPV1 is located in the regions that modulate nociception and autonomic functions in the spinal cord^[^^31^^]^. TRPV1 and MOR (morphine receptors) are localized in DRG in the spinal cord^[^^33^^]^. Many studies have indicated that TRPV1 channels could be regulated by MOR^[^^1^^,^^35^^,^^36^^]^. The present study revealed that hyper-stimulation of MOR in chronic administration of morphine could upregulate TRPV1 channels. In another study, reduction of TRPV1 expression in sensory neurons by RTX attenuated the development of morphine tolerance and altered the presynaptic effects of the MOR agonist in the spinal cord^[^^34^^]^. Furthermore, TRPV1 antagonists reversed both thermal and mechanical morphine-induced hyperalgesia^[^^37^^]^. Studies have indicated that allodynia and hyperalgesia induced by morphine tolerance could be modulated by the inhibition of TRPV1 channels^[^^32^^]^. The present study confirmed that TRPV1 plays an important role in the development of morphine tolerance, and baclofen can modify TRPV1 channel expression. GABA_B _receptor is expressed in close proximity to TRPV1 and colocalizes at nociceptor endings^[^^14^^]^. Baclofen could reduce hyper-sensitization, hyperalgesia, and allodynia in morphine tolerance by modulating TRPV-1 channels. GABA_B_ agonist may modulate TRPV1 channel by modulating PLC/PKC pathways that result in the sensitization of the capsaicin receptor^[^^14^^]^.

Upregulation of TRPV1 channels can activate many signaling pathways, such as cAMP/PKA, β-arrestin, and MAPK^[^^1^^]^. The present study also demonstrated that the upregulation of TRPV1 by morphine tolerance phenomena can activate PKC pathway. Another study has demonstrated that PKC can activate more TRPV1 channels as a positive feedback and hyper-sensitize sensory neurons^[^^27^^]^. In fact, PKC phosphorylation sensitizes TRPV-1 channel, but it fails to activate the TRPV-1 channel directly^[^^24^^]^. TRPV1 function enhanced in murine DRG neurons via TRPV1 phosphorylation by PKCɛ^[^^38^^]^. Phosphorylation by PKC sensitizes the channel to capsaicin, protons, and heat and can induce hyperalgesia in rats^[^^39^^,^^40^^]^. Phosphorylating TRPV1 at different sites might exert varied functional consequences. Differential phosphorylation by PKC and PKA mediates different types of TRPV1 gating. PKA can be activated by TRPV1 channel and PKA with a negative feedback mechanism by phosphorylation of an exclusive site can desensitize TRPV1 channel^[^^41^^]^. Previous surveys have indicated that GABA_B_ agonists have no effect on cAMP/PKA pathway for TRPV1 regulation^[42,43]^; however, a number of studies have displayed that baclofen can modulate this receptor by PLC/PKC pathways and regulate its functions. GABA_B_ receptors trigger a wide range of cellular responses. Otherwise, the PLC/PKC is not the sole mediator for baclofen to regulate TRPV-1 functions. Rather, it is possible that other mechanisms are targeted by baclofen to protect TRPV1 from overexpression and hyper-sensitivity in morphine tolerance and dependence. 

In this study, evidence provided that baclofen as a GABA_B_ agonist can modulate changes in DRG in morphine tolerance. One of these changes was TRPV1 over-expression and TRPV1 channel hypersensitivity by PKC over-expression in DRG. Baclofen limits TRPV1 activity by modulating TRPV1 expression, and PLC/PKC pathway could enhance anti-nociceptive effect of morphine in behavioral studies and reduce hypersensitivity and hyperalgesia induced by TRPV1 hyperactivity in chronic morphine administration.
